# Effects of Lactic Acid Bacteria and Molasses Additives on Dynamic Fermentation Quality and Microbial Community of Native Grass Silage

**DOI:** 10.3389/fmicb.2022.830121

**Published:** 2022-03-16

**Authors:** Yuyu Li, Shuai Du, Lin Sun, Qiming Cheng, Junfeng Hao, Qiang Lu, Gentu Ge, ZhiJun Wang, Yushan Jia

**Affiliations:** ^1^Key Laboratory of Forage Cultivation, Processing and High Efficient Utilization of Ministry of Agriculture and Rural Affairs, Inner Mongolia Agricultural University, Hohhot, China; ^2^Key Laboratory of Grassland Resources of Ministry of Education, Inner Mongolia Agricultural University, Hohhot, China; ^3^National Engineering Laboratory of Biological Feed Safety and Pollution Prevention and Control, Key Laboratory of Animal Nutrition and Feed Science of Zhejiang Province, Institute of Feed Science, Zhejiang University, Hangzhou, China; ^4^Inner Mongolia Academy of Agricultural & Animal Husbandry Sciences, Hohhot, China; ^5^College of Animal Science, Guizhou University, Guiyang, China

**Keywords:** native grass, lactic acid bacteria, molasses, microorganisms, silage

## Abstract

Ensiling native grass is an effective method to protect the nutritional quality of forage and alleviate feed shortages in the cold winter of the Inner Mongolian Plateau. To improve the usability of native grass resources as feed in China, the effects of lactic acid bacteria and molasses additions on the microbial population, fermentation quality, and nutritional quality of native grass during silage were investigated. Treatments were a control treatment with no additive (CK), lactic acid bacteria (L), molasses (M), and lactic acid bacteria in combination with molasses (L+M), all of which were stored at ambient temperature (17–28°C) for 7, 14, 30, and 60 days. The results showed that all additives improved nutritional value and fermentation quality with low pH and ammonia nitrogen (NH_3_–N) and high crude protein (CP) and water soluble carbohydrate (WSC) than control silage over the ensiling period. Compared with L or M silage, the L+M silage combination improved fermentability, as evidenced by higher LA content and a faster pH drop during the first 7 days of ensiling. With prolonged ensiling time, the combined addition of L and M could increase the count of desirable *Lactobacillus*, decrease microbial diversity, and inhibit the growth of undesirable microorganism, such as Clostridia, *Escherichia*, and *Enterobacter* abundance compared with silage treated with CK, L. or M. Application of L together with M could further improve the silage quality of native grass by altering bacterial community structure. In summary, the addition of lactic acid bacteria and molasses increased the relative abundance of *Lactobacillus* of native grass silage and improved fermentation quality.

## Introduction

Native grass is an herb that grows on native grasslands, which are widely scattered across the Mongolian Plateau; large needlegrass (*Stipa grandis* P. Smirn) and Chinese leymus [*Leymus chinensis* (Trin.) Tzvel] dominate typical steppe communities (Zhang et al., 2016), are mostly utilized for grazing and haymaking, and are the main source of forage in pastoral areas (Du S. et al., 2020). Seasonal changes affect the quality and productivity of native grass (Kang et al., 2007). From June to September, native grass grows well, and animals usually get enough nutrition and gain weight during this time (Long et al., 1999). However, feed deficiency during the long cold season (November to June) is one of the major issues confronting the Mongolian Plateau’s traditional animal grazing system (Zhou et al., 2017; Ding et al., 2020), which is due to both the limited quantity and quality of native grass during this period. Farmers usually begin storing grass in mid-August. During harvest and storage, there is a significant loss of dry matter (DM) and crude protein (CP) (Scarbrough et al., 2002). Therefore, efficient native grass preservation is regarded as a crucial farming strategy for alleviating feed shortages in the Mongolian Plateau’s traditional year-round grazing system.

Ensiling has been considered as a traditional method to preserve forage nutrients in the harvesting season because of various advantages, such as easy to operate, prolonging supply time, and suitability for a wide range of feedstock (Ding et al., 2020), and can effectively address animal feed shortages during the winter months on the Mongolian Plateau (Zhang et al., 2016). Ensiling mainly depends on lactic acid bacteria to transfer soluble carbohydrates to lactic acid, resulting in a low pH environment and inhibiting the growth of aerobic bacteria, making the feed preserved (Lee et al., 2018) and improving palatability (Driehuis, 2013). Ensiling has been shown in previous research to improve fermentation quality while also preserving the nutrients of natural grass, resulting in a high feeding value (Khota et al., 2016; Du Z. M. et al., 2020). However, due to the low water content, low water soluble carbohydrate, low lactic acid bacteria attachment, and high buffer energy of natural forage, direct silage is difficult to succeed (Zhang et al., 2016; Hou et al., 2017). To solve this problem, Cai et al. (2020) evaluated the effects of additives on native grass silage and found that the use of L and cellulase can improve the fermentation quality of native grass silage. L is the most common type of silage additive, capable of ensuring extensive fermentation and efficient substrate utilization in ensiled materials. It has been reported that adding L to rice straw silage improved its quality (Zhao et al., 2019). M is a sugar industry byproduct that is high in soluble carbohydrate and serves as a fermentable substrate. M not only improved fermentation quality but also significantly altered the microbial community of cassava foliage silage (Li et al., 2021). However, there is limited information related to the microbial community and fermentation products during the ensiling process of native grass with additive treatments. Therefore, this study was conducted to increase understanding to inform the regulation of fermentation of native grass on the Mongolian Plateau. The purpose of this study was to assess the effects of adding L, M, and their combination on the fermentation quality and microbial community dynamics of native grass silage.

## Materials and Methods

### Study Sites and Silage Preparation

Native grass was harvested at the heading stage from a typical grassland (45°58′E, 118°57′N) on the Inner Mongolian Plateau, People’s Republic of China, on July 15, 2020. The dominant species harvested were large needlegrass (*Stipa grandis* P. Smirn), Chinese Leymus [*Leymus chinensis* (Trin.) Tzvel.], and Dahurian Bushclover [*Lespedeza davurica* (Laxm.) Schindl.]. Harvested native grass was chopped to lengths of 2–3 cm. Lactic acid bacteria (L) inoculant and molasses (M) were used as additives for ensiling the native grass. L (*Lactobacillus plantarum*, Snow Brand Seed Co. Ltd., Sapporo, Japan) was provided by the Institute of Animal Husbandry and Grassland of Japan. M was obtained from Baihui Biological Technology Co. Ltd. (Chifeng, Inner Mongolia, China). The L inoculant was applied at a level of 10^6^ colony-forming units (cfu) per gram of fresh material (FM), and molasses was applied at 30 g/kg of FM. The chopped native grass was mixed and divided into equal portions for four treatments: no additive control (CK), L treatment (L), M treatment (M), and L + M treatment (L + M). All the additives were mixed homogenously with native grass. In detail, each treated batch was divided into four replicates (one for backup) of 200 g each, which were packed into polyethylene bags and vacuum sealed. A total of 64 bags (4 treatments × 4 ensiling days × 4 repeats) were prepared and kept at ambient temperature (17–28°C). After 7, 14, 30, and 60 days of ensiling, fermentation characteristics, chemical compositions, microorganism counts, and the microbial community were analyzed. Initial fresh forage samples were taken before ensiling.

### Analysis of Chemical Composition, Organic Acid, and Microbial Population

For chemical analysis, each sample set had three parallel determinations. Dry matter (DM) contents were determined by oven drying at 65°C for 48 h (Zhang et al., 2016). The crude protein (CP) content was computed by multiplying TN content by 6.25 (Sun et al., 2021). The neutral detergent fiber (NDF) and acid detergent fiber (ADF) contents of fresh material were determined by the methods of Van Soest et al. (1991). The WSC was determined using the method of Thomas (1977).

Each polyethylene bag was opened on a clean bench. About 10 g of fresh forage or silage was blended with 90 ml of sterilized water, and the extract was serially diluted to quantify the bacterial group in a sterile solution. The numbers of LAB, aerobic bacteria, coliform bacteria, yeast, and molds were measured through the method of Sun et al. (2021).

Last, the liquid extract was filtered through four layers of cheesecloth and filtered paper. The prepared filtrate was used to analyze the pH values, organic acids, and ammonia nitrogen (NH_3_–N) of the sample. The lactic acid (LA), acetic acid (AA), propionic acid (PA), and butyric acid (BA) content of silage was analyzed by high-performance liquid chromatography according to the method of Cheng et al. (2021). The nitrogen (NH_3_–N) content was determined according to the method of Broderick and Kang (1980).

### DNA Extraction, Polymerase Chain Reaction Amplification, and Sequencing

The E.Z.N.A.^®^ sample DNA kit was used to isolate bacterial community genomic DNA from native grass and silage samples. The reagent, which was developed to extract DNA from trace amounts of sample, has been demonstrated to be effective for preparing DNA from most bacteria. The blank was made of non-nuclear water. Total DNA was eluted in 50 L of elution buffer and stored at −80°C until it was measured in the PCR (LC-Bio Technology Co. Ltd., Hangzhou, Zhejiang Province, China).

Subsequently, extracted DNA samples were subjected to PCR amplification on bacteria 16S rDNA (V3 and V4 regions). For bacterial amplification, the following primers were used: 341F and 805R. The PCR reactions were conducted using the following program: 3 min of denaturation at 95°C, 27 cycles of 30 s at 95°C, 30 s for annealing at 55°C, and 45 s for elongation at 72°C, and a final extension at 72°C for 10 min. PCR reactions were performed in triplicate 20-μl mixture containing 4 μl of 5× FastPfu buffer, 2 μl of 2.5 mM dNTPs, 0.8 μl of each primer (5 μM), 0.4 μl of FastPfu Polymerase, and 10 ng of template DNA. Agarose gel electrophoresis [2% (w/w)] was used to confirm the PCR products. To eliminate the possibility of false-positive PCR results as a negative control, ultrapure water was used throughout the DNA extraction process instead of a sample solution. AMPure XT beads (Beckman Coulter Genomics, Danvers, MA, United States) were used to purify the PCR products, and Qubit was used to quantify them (Invitrogen, Carlsbad, CA, United States).

The PCR products were sequenced on an Illumina platform (Guangzhou Gene Denovo Co. Ltd., Guangzhou, China) after purification and quantification. Fqtrim was used to perform quality filtering on the raw reads under specific filtering conditions in order to obtain high-quality clean tags (v0.94). Vsearch software (v2.3.4) was used to filter chimeric sequences. QIIME2 was used to calculate alpha and beta diversity, with the same number of sequences extracted randomly by reducing the number of sequences to the minimum of some samples, and relative abundance (X bacteria count/total count) was used in bacteria taxonomy. The QIIME2 process was used to examine alpha and beta diversity. Blast was used to perform species annotation sequence alignment, and the alignment databases were SILVA and NT-16S.

### Statistical Analyses

The data was subjected using two-way analysis of variance with the fixed effects of additives, ensiling time, and additives × ensiling time using the general linear model (GLM) procedure of SAS 9.3 software (SAS Institute, Inc., Cary, NC, United States). The effect was considered significant when *p* < 0.05. The data of high-throughput sequencing were analyzed on the free online platform www.omicstudio.cn.

## Results

### Chemical Parameters and Microbial Compositions of Silage Materials

The chemical parameters and microbial compositions by native grass before ensiling are presented in Table 1. The DM, WSC, CP, NDF, and ADF were 37.72, 4.31, 10.83, 62.98, and 36.58%, respectively. Microbial compositions in the native grass for L, aerobic bacteria, coliform bacteria, and yeasts were 4.26, 8.75, 5.82, and 6.35 log_10_ cfu/g FM, respectively. Fresh native grass molds were below the detectable level.

**TABLE 1:** Chemical and microbial compositions of fresh native grass.

	Items	*Sample*	*SEM*
Chemical composition	DM (%FM)	37.72	2.38
	WSC (%DM)	4.31	0.03
	CP (%DM)	10.83	0.43
	NDF (%DM)	62.98	3.03
	ADF (%DM)	36.58	1.07
Microbial counts	L (log cfu/g FM)	4.26	0.11
	Aerobic bacteria (log cfu/g FM)	8.75	0.03
	Coliform bacteria (log cfu/g FM)	5.82	0.15
	Yeast (log cfu/g FM)	6.35	0.29
	Molds (log cfu/g FM)	ND	ND

*FM, fresh material; DM, dry matter; CP, crude protein; NDF, neutral detergent fiber; ADF, acid detergent fiber; WSC, water-soluble carbohydrates; L, lactic acid bacteria; ND, not detected, SEM, standard error of the mean.*

### Effect of Additives and Ensiling Days on Chemical Parameters of Native Grass Silage

Effects of additives and ensiling days on chemical parameters of native grass silages are shown in Table 2. The additive treatments significantly altered the contents of DM, CP, and WSC, but had no effects on NDF and ADF contents. L + M silage had higher WSC and CP contents and lower ADF and DNF contents compared with L and M silage after 60 days of silage. Ensiling days had significant (*p* < 0.05) effects on the chemical composition (except DM) of silage. All additives, except for the CK treatment, significantly decreased (*p* < 0.05) NDF content of native grass silage. In addition, the L + M silage had significantly (*p* < 0.05) higher CP contents than other silage at the end of ensiling. The WSC contents in M and M + L silages were significantly (*p* < 0.05) lower than the CK silage. The NDF and ADF content exhibited a continuous downtrend during the whole ensiling process. The interaction of additive treatments and ensiling days had a significant impact on silage WSC (*p* < 0.05), but did not affect the silage DM, CP, NDF, or ADF contents (*p* > 0.05).

**TABLE 2:** Effect of additives and ensiling days on chemical compositions of native grass silages.

Items	Treatment	Ensiling days				Significance			
		7	14	30	60	SEM	*T*	*D*	*T* * *D*
DM (%FM)	CK	35.97aA	35.79bA	34.72bAB	34.63cB	0.13	**	NS	NS
	L	36.37aA	35.92bAB	35.33bB	35.19abB				
	M	36.16aA	35.47bAB	35.06bB	35.02bcB				
	L + M	37.19aA	36.62aA	36.17aA	36.01aA				
CP (%DM)	CK	9.71aA	8.97bAB	8.49bBC	7.87bC	0.11	**	**	NS
	L	10.07aA	9.67aA	9.34aAB	8.57bB				
	M	10.26aA	9.91aA	9.17abB	8.58bB				
	L + M	10.48aA	9.86aB	9.65aB	9.08aC				
NDF (%DM)	CK	61.11aA	58.03aA	57.63aA	57.45aA	0.42	NS	**	NS
	L	59.43aA	58.66aAB	57.73aAB	56.46aB				
	M	59.77aA	58.87aA	56.11aB	55.52aB				
	L + M	57.70aA	55.92aB	54.45aB	53.92aC				
ADF (%DM)	CK	33.79aA	31.34aA	31.90aA	31.04aA	0.33	NS	*	NS
	L	32.58aA	32.32aA	30.58aA	30.47aA				
	M	32.72aA	31.69aA	29.99aA	29.08aA				
	L + M	31.01aA	30.37aA	29.53aA	28.26aB				
WSC (%DM)	CK	3.78bA	3.04cB	2.87bBC	2.54cC	0.08	**	**	*
	L	3.95abA	3.17bcB	3.10bB	2.82bcB				
	M	4.26abA	3.72abB	3.40aBC	3.20abC				
	L + M	4.34aA	3.98aB	3.63aC	3.50aC				

FM, fresh material; DM, dry matter; CP, crude protein; NDF, neutral detergent fiber; ADF, acid detergent fiber; WSC, water-soluble carbohydrates; LAB, lactic acid bacteria; ND, not detected; CK, no additive control; L, lactic acid bacteria; M, molasses; L + M, lactic acid bacteria + molasses; SEM, standard error of the mean; T, treatments; D, ensiling days; T * D, interaction between treatments and ensiling days. *Significant at 0.05. **Significant at 0.01. Means in the same column (a–c) or row (A–C) with different superscript letters differ significantly from each other (p < 0.05).

### Effect of Additives and Ensiling Days on Fermentation Quality of Native Grass Silage

Table 3 illustrates the dynamics of the fermentation quality of native grass silage during ensiling. These parameters were significantly affected by additive treatments, ensiling days, and their interaction (*P* < 0.05). Compared with the control, all additives significantly (*p* < 0.05) increased lactic acid (LA) concentration, and decreased acetic acid (AA), propionic acid (PA), and NH_3_–N concentration in silage. Compared with the other treatments, L + M addition further increased LA concentration and decreased the contents of AA, PA, and NH_3_–N; butyric acid (BA) was not detected in the native grass silage. The highest LA concentration was recorded in L + M silage, which had a value of 29.94% on the 60th day of ensiling (*p* < 0.05). The pH values of the additive silages were significantly decreased (*p* < 0.05) compared with the control during 14 days of ensiling, and L + M silage always maintained a lower pH value during ensiling. Moreover, NH_3_–N content exhibited a continuous increasing trend during the whole ensiling process, and the content of NH_3_–N in the L + M treatment group remained low after 14 days of ensiling.

**TABLE 3:** Effect of additives and ensiling days on fermentation quality of native grass silages.

Items	Treatment	Ensiling days				Significance			
		7	14	30	60	SEM	*T*	*D*	*T* * *D*
pH value	CK	4.66aA	4.63aA	4.48aB	4.32aC	0.04	**	**	**
	L	4.54aA	4.14bB	4.05bB	4.03cB				
	M	4.63aA	4.50aAB	4.53aAB	4.25bB				
	L + M	4.18bA	4.01bB	3.95bBC	3.91dC				
LA (%DM)	CK	9.17bD	11.05cC	14.76cB	17.47cA	0.88	**	**	**
	L	13.08aD	13.34bC	19.49bB	26.61bA				
	M	12.3aD	13.82bC	17.3bB	23.74bA				
	L + M	14.64aD	21.76aC	26.00aB	29.94aA				
AA (%DM)	CK	2.26aD	5.38aC	10.86B	14.75aA	0.56	**	**	**
	L	1.16bD	3.18bC	5.39cB	7.61cA				
	M	2.67aD	3.28bC	6.65bB	11.49bA				
	L + M	1.09bD	2.34cC	3.51dB	5.92dA				
PA (%DM)	CK	0.97aD	1.79aC	2.91aB	4.89aA	0.17	**	**	**
	L	0.56bD	1.45bC	2.52bB	2.96bA				
	M	0.88aC	1.58abBC	1.96cB	3.57bA				
	L + M	0.3cD	1.42cC	1.91cB	2.65bA				
NH_3_–N (%TN)	CK	1.39aB	2.01aB	2.87aB	6.05aA	0.18	*	**	**
	L	1.22aC	1.44aC	2.19aB	3.33bA				
	M	1.05aC	1.52aBC	2.03aB	2.69bA				
	L + M	1.12aC	1.42aBC	1.81aB	2.3bA				

LA, lactic acid; AA, acetic acid; PA, propionic acid; NH_3_–N, ammonia nitrogen; TN, total nitrogen; ND, not detected; SEM; ND, no detected; CK, no additive control; L, lactic acid bacteria; M, molasses; L + M, lactic acid bacteria + molasses; SEM, standard error of the mean; T, treatments; D, ensiling days; T * D, interaction between treatments and ensiling days. *Significant at 0.05. **Significant at 0.01. Means in the same column (a–d) or row (A–D) with different superscript letters differ significantly from each other (p < 0.05).

### Effect of Additives and Ensiling Days on Microorganism Counts of Native Grass Silage

Table 4 shows the effects of additives and ensiling days on microbial counts of native grass silages. Overall, additive treatments, ensiling days, and their interaction had a significant impact on microbial counts (*p* < 0.05). Additive treatment silages had lower (*p* < 0.05) coliform bacteria content and higher L and yeast numbers compared with the control silage after 60 days of ensiling. Compared with other treatments, the L and yeast contents in L + M-treated silage were significantly (*p* < 0.05) higher, while the coliform bacteria contents were lower than that of other silages at the end of ensiling. The microbial count of silages exhibited a continuous decreasing trend during the whole ensiling process. Mold counts were not detected in any treatment after 14 days of ensiling.

**TABLE 4:** Effect of additives and ensiling days on microorganism counts of native grass silages.

Items	Treatment	Ensiling days				Significance			
		7	14	30	60	*SEM*	*T*	*D*	*T* * *D*
L (log cfu/g FM)	CK	8.74cA	8.07aB	6.50bC	5.74cD	0.16	**	**	**
	L	8.99abA	8.16aB	6.81abC	6.56bD				
	M	8.89bcA	8.06aB	6.45bC	6.45bC				
	L + M	9.09aA	8.29aB	7.00aC	6.77aC				
Yeast (log cfu/g FM)	CK	6.37dA	5.08bB	3.35cC	3.38cC	0.28	**	**	**
	L	8.84bA	7.06aB	3.51cD	3.91bC				
	M	6.92cA	7.54aA	3.84bB	3.50bcB				
	L + M	9.35aA	6.99aB	5.91aC	5.62aD				
Aerobic bacteria (log cfu/g FM)	CK	6.38aA	5.68aA	3.67aB	3.63aB	0.15	**	**	*
	L	4.73abA	3.45bB	3.23bB	3.16bB				
	M	4.53bA	3.49bB	3.53aB	3.30bB				
	L + M	3.66bA	3.38bB	3.17bB	2.62cC				
Coliform bacteria (log cfu/g FM)	CK	5.76a	ND	ND	ND	0.29	**	**	**
	L	3.62b	ND	ND	ND				
	M	3.95b	ND	ND	ND				
	L + M	2.85c	ND	ND	ND				

CK, no additive control; L, lactic acid bacteria; M, molasses; L + M, lactic acid bacteria + molasses; ND, no detected; SEM, standard error of the mean; T, treatments; D, ensiling days; T * D, interaction between treatments and ensiling days. *Significant at 0.05. **Significant at 0.01. Means in the same column (a–d) or row (A–D) with different superscript letters differ significantly from each other (p < 0.05).

### Microbial Community of Native Grass Silage

The bacterial diversity of native grass silage was discovered using high-throughput analyses targeting variable regions 3 and 4 of 16S rDNA. The valid sequences of all 51 triplicate samples added up to 1,294,499 after unqualified sequences were removed. The average Good’s coverage for all of the samples was greater than 99%, indicating that the sequencing depth was sufficient for reliable bacterial community analysis (Table 5). The interaction of additive treatments and ensiling days had a significant impact on Simpson and Shannon (*p* < 0.05), but did not affect Reads, OUT, and Chao1 (*p* > 0.05). According to the OTU and Chao 1 indexes, the bacterial community’s richness decreased with increased ensiling time in the CK silage. The Shannon and Simpson indexes were remarkably lower in the M and L + M-treated silages than in the CK silage at days 7 and 14 of ensiling (*p* < 0.05). In addition, the Chao 1 index and Simpson index showed trends similar to the trend in OTUs. Compared with other treatment groups, L + M addition resulted in the decrease in the richness of the bacterial community without dose effect in silage at day 60 of ensiling (*p* > 0.05). The lowest Shannon index of bacterial diversity was observed in the L + M-treated silage (i.e., 3.01).

**TABLE 5:** Effect of additives and ensiling days on bacterial alpha diversity of native grass silages.

Items	Treatment	Ensiling days				Significance			
		7	14	30	60	*SEM*	*T*	*D*	*T* * *D*
Reads	CK	72825aA	77336aA	75397aA	77922aA	407.39	NS	NS	NS
	L	75263aA	75315aA	77059aA	78374aA				
	M	75654aA	78011aA	77415aA	75825aA				
	L + M	76901aA	75853aA	77805aA	78222aA				
OUT	CK	254aA	181aA	189aA	117aA	12.53	NS	NS	NS
	L	117aA	135aA	135aA	199aA				
	M	88aA	136aA	133aA	134aA				
	L + M	118aA	127aA	235aA	115aA				
Chao1	CK	271.9aA	195.0aA	193.2aA	119.0aA	13.71	NS	NS	NS
	L	119.0aA	142.0aA	141.3aA	204.9aA				
	M	91.2aA	138.4aA	136.2aA	136.1aA				
	L + M	120.3aA	134.2aA	249.3aA	118.6aA				
Simpson	CK	4.05aA	4.34aA	4.46aA	2.94aB	0.09	**	**	*
	L	3.30abA	3.72abA	3.57aA	3.72aA				
	M	2.77bA	3.36bA	3.55aA	3.66aA				
	L + M	2.96bA	3.28bA	3.63aA	3.01aA				
Shannon	CK	0.86aA	0.88aA	0.90aA	0.67aA	0.01	NS	*	*
	L	0.79abA	0.84aA	0.82aA	0.83aA				
	M	0.77bA	0.82aA	0.84aA	0.87aA				
	L + M	0.77bA	0.83aA	0.84aA	0.76aA				
Coverage	CK	0.9988aA	0.9995aA	0.9997aA	0.9998aA	0.0001	NS	NS	NS
	L	0.9998aA	0.9997aA	0.9997aA	0.9995aA				
	M	0.9998aA	0.9997aA	0.9997aA	0.9998aA				
	L + M	0.9997aA	0.9996aA	0.9991aA	0.9997aA				

CK, no additive control; L, lactic acid bacteria; M, molasses; L + M, lactic acid bacteria + molasses; ND, no detected; SEM, standard error of the mean; T, treatments; D, ensiling days; T * D, interaction between treatments and ensiling days. *Significant at 0.05. **Significant at 0.01. Means in the same column (a,b) or row (A,B) with different superscript letters differ significantly from each other (p < 0.05).

**FIGURE 1:**
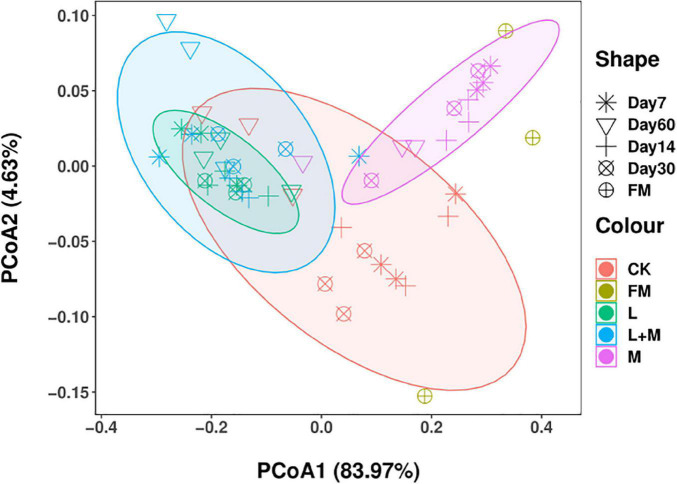
The community dissimilarities in different additives treatments and fermentation time, calculated via weighted UniFrac distances, with coordinates calculated using principal coordinates analysis (PCoA). FM, fresh material; CK, no additive control; L, lactic acid bacteria; M, molasses; L + M, lactic acid bacteria + molasses.

The PCoA analysis (Figure 1) showed the differences in bacterial communities between fresh samples and different additive treatments. The bacterial communities of fresh samples and different additive treatments were significantly separated, indicating that the microbial communities of fresh samples and different additive treatments changed during silage. A clear difference in the additive treatments in contrast with the control indicates that the additive treatments and control silage bacterial communities were different on day 60 of ensiling.

**FIGURE 2:**
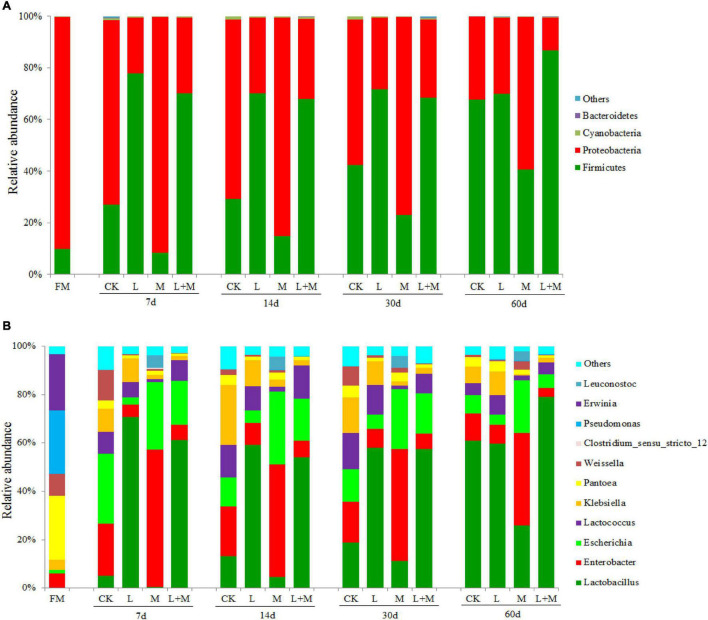
The bacterial abundance at phylum and genus level in native grass silage. FM, fresh material; CK, no additive control; L, lactic acid bacteria; M, molasses; L + M, lactic acid bacteria + molasses. The bacterial communities are shown at the phylum level **(A)** and the genus level **(B)**.

The dynamics of bacterial communities in native grass silage at the phylum level are shown in Figure 2A. *Proteobacteria* (89.82%) and *Firmicutes* (9.87%) dominated the majority of the total phylum sequence in the fresh sample. During the process of ensiling, *Firmicutes* and *Proteobacteria* dominated in all groups, but the community composition was affected by the ensiling treatments. Compared with the CK treatment, *Firmicutes* increased and *Proteobacteria* decreased in the L and L + M-treated groups. However, the relative abundance of *Proteobacteria* in M treatments was higher than that of the other treatments. In addition, the increase in *Firmicutes* abundance was greater with added L + M, and the relative abundance of *Firmicutes* (at 60 days) was higher.

Figure 2B illustrates the dynamics of bacterial populations in native grass silage at the genus level. The main epiphytic genera of fresh native grass were *Pantoea* and *Pseudomonas*, followed by *Erwinia*. Their abundance decreased greatly during the ensiling process, especially in the treated silage. *Lactobacillus* was the most abundant genus in silage with *L. plantarum* added (L and L + M; >54%) after 30 days of ensiling. The addition of L and L + M decreased the abundance of *Enterobacter* and *Escherichia* compared with silage with added M alone. In the absence of additives, after 60 days of ensiling, the dominant species were *Pantoea* and *Lactobacillus*. Under the influence of the fermentation promoter, there was a slight difference from the CK treatment. The main microbes were *Lactobacillus* (60.96%) and *Enterobacter* (11.26%) in the control group, whereas the main genera were *Lactobacillus* (58.29%), *Enterobacter* (7.72%), *Escherichia* (4.00%), and *Pantoea* (4.17%) in the L group. In the M group, the main genera were *Lactobacillus* (23.86%), *Enterobacter* (35.31%), *Escherichia* (20.12%), and *Pantoea* (1.74%), and the main genera were *Lactobacillus* (67.44%), *Escherichia* (4.85%), and *Enterobacter* (3.29%) in the L + M combination group.

**FIGURE 3:**
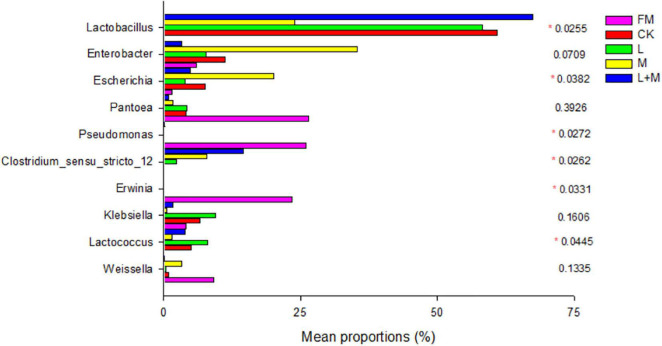
One-way analysis of variance bar plots of the genus level (10 most abundant genera) among native grass silage groups after 60 days of silage. *p < 0.05. FM, fresh material; CK, no additive control; L, lactic acid bacteria; M, molasses; L + M, lactic acid bacteria + molasses.

To further reveal the effect of additives on the bacterial community in natural forage silage, one-way analysis of variance bar plots of the genus level among native grass silage groups after 60 days of ensiling are shown in Figure 3. In this study, *Pantoea* was the dominant genus in the fresh native grass (FM) (26.48%). Furthermore, *Clostridium_sensu_stricto_*12 was the subdominant family in FM, following *Pantoea*. The additive treatments significantly changed the relative abundance of *Lactobacillus*, *Enterobacter*, *Escherichia*, *Pantoea*, *Pseudomonas*, *Clostridium_sensu_stricto_*12, *Erwinia*, *Klebsiella*, and *Lactococcus* after 60 days of native grass silage. Lactobacillus was the most abundant genus in the L + M-treated silage (L + M; >65%). Molasses enhanced the growth of *Enterobacter* and *Escherichia*.

**FIGURE 4:**
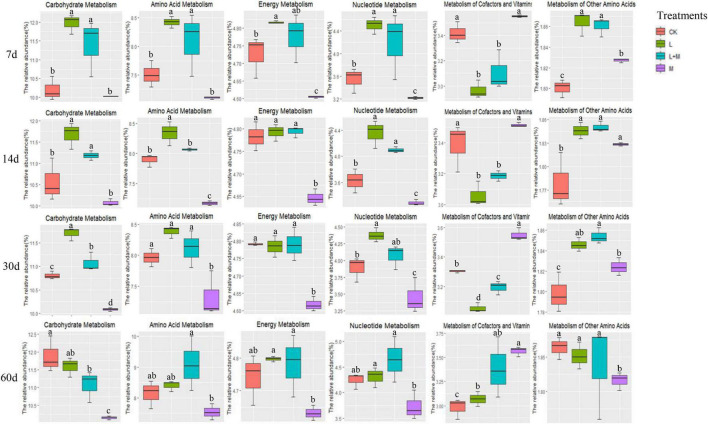
Statistics of additives and ensiling days on the abundance of native grass silage microorganism KEGG pathway. Summary of significant functional shifts predicted using Phylogenetic Investigation of Communities by Reconstruction of Unobserved States (PICRUSt). For each KEGG pathway, the second level of the predicted functional shift is shown with respect to the fermentation processes and additive treatments. a–d indicates significant differences between different additive treatments in the same silage period at *p* < 0.05. CK, no additive control; L, lactic acid bacteria; M, molasses; L + M, lactic acid bacteria + molasses.

The effects of metabolic pathway changes in bacterial communities were quantified by predicting the functional characteristics of bacterial communities. Therefore, Figure 4 showed the metabolic pathways of silage treated with different additives. In the present study, higher relative abundance of carbohydrate metabolism was observed in the L-inoculated silage during the early phase of ensiling (7 days). With increased fermentation time, the relative abundance of carbohydrate metabolism gradually increased. Amino acid metabolism was lowest in the M-inoculated silage from 7 days until the end of ensiling. Metabolism of other amino acids showed the same pattern. The relative abundance of energy metabolism in L and L + M silages were higher than that of the CK and M treatments during the entire ensiling period, and nucleotide metabolism showed the same trend. After 60 days of fermentation of silage treated with additives, the metabolism of cofactors and vitamins were increased.

## Discussion

The amount of epiphytic L to raw materials is the key factor for the success of silage (Cai et al., 1999). The amount of epiphytic L is more than 10^5^ cfu g^–1^ of FM; it is easier for silage to be successful and preserved (Cai et al., 1999). In addition, WSC is a significant factor for silage fermentation; the content of WSC in raw material is more than 5% DM, which can ensure the success of silage fermentation (Moselhy et al., 2015; Ni et al., 2017). The content of WSC and the number of epiphytic L influence the rate of increase in LA and drop in pH during the early stages of ensiling, which are critical for successful silage (Wang S. et al., 2019). In our study, the counts of L on native grass were <10^5^ cfu g^–1^ of FM (Table 1), and the WSC content of FM was 4.31, which indicated difficulty in good preservation of native grass through ensiling without any additives. Thus, it was necessary to add exogenous L and/or M to enhance lactic acid fermentation during ensiling.

Silage treated with L + M had higher content of DM than that of other silages and could be attributed to molasses providing additional substrates, promoting sufficient lactic acid production, and resulting in pH reduction and DM loss prevention (Muck et al., 2018); this finding is similar to that of Chen et al. (2017). The addition treatment silages had lower NDF and ADF content than the control silages, which might be due to the additives encouraging microorganism proliferation and enhancing microbial respiration and fermentation of the fiber component. This finding is in line with those of Baytok et al. (2014) and Li et al. (2021). During the ensiling process of native grass, the content of CP significantly decreased. This was mostly due to the fact that some microorganisms involved in silage fermentation degrade protein (Du Z. M. et al., 2020). Sufficient content of WSC could provide more substance for lactic acid fermentation. The study of Ge et al. (2018) found that inoculating with L would ensure efficient conversion and utilization of WSC during ensiling, followed by a considerable drop in pH and reduction of nutrient loss, because L transformed the WSC into lactic acid during the early stage of fermentation. Therefore, the addition of M and L prior to ensiling seems to be necessary for high-quality native grass silage.

Ensiling is a complex bacterial fermentation process that causes a buildup of organic acid and a drop in pH (Ding et al., 2020). Silage pH is a key indication for determining fermentation quality, with a pH of 4.2 or below indicating well-fermented silage (Mcdonald et al., 1991). All of the treatments in our research had lower pH values than the CK treatment for the whole ensiling time, and a combination of L and M had the highest reduction in pH among the treatments. In this paper, it was found that the decrease in pH value occurred mainly in the first 7 or 14 days of silage, and then decreased with the increase in silage time, which was following the report of Ni et al. (2017). After 60 days of ensiling, the pH values of the L + M treatments declined to below 4.00, which could be due to the direct increase in fermentable substrate that promoted sufficient LA production and preserved silage due to the low pH, and which may also explain the decreased concentration of PA. In contrast, the pH value of the CK treatment remained above 4.20. The above results further showed that the addition of L and M prior to ensiling could stimulate the decrease in pH. In addition, L addition showed lower pH values than with M addition alone, which may be because the WSC were decomposed to a certain extent by L in the early ensiling stage, which, in turn, might accelerate the domination of L (Wang et al., 2021). Our present study showed that additive treatments decreased pH, AA, PA, and NH_3_–N contents, as a result of the increased LA contents in silage. The L and L + M silages had higher LA contents than other silage treatments. This may be because both additives contained L, which could initiate an accumulation of LA, thereby resulting in a decrease in the final pH of silage (Martinez-Fernandez et al., 2010). This is in line with studies on typical woody forage (Wang et al., 2021), fodder ramie (Ning et al., 2012), and soybean (Ni et al., 2017). NH_3_–N production is related to CP degradation in all silages and reveals the extent of proteolysis in silage (Wilkinson, 2005). Compared with the CK silage, adding L and M alone or in combination significantly decreased NH_3_–N content in silage, which might be because the addition of additives reduces the pH value, and the acidic environment reduced undesirable fermentation and proteolysis. After 60 days of ensiling, the decline in NH_3_–N content in L and L + M might be because some LAB can induce nitrification, which transformed ammonia nitrogen to nitrate nitrogen (Si et al., 2019). Similarly, Hou et al. (2017) found that additive treatments improved the fermentation quality of native grass silage. The abovementioned results indicated that L and M addition in the ensiling process could promote fermentation quality, and that the combination treatment enhanced the fermentation more efficiently.

Before ensiling, L, aerobic bacteria, coliform bacteria, molds, and yeasts are often found in Inner Mongolian native grasses. Aerobic bacteria are the dominating microorganisms during the initial stage of ensiling, resulting in some fermentation loss (Ge et al., 2018). In the present study, L counts of all silages during ensiling were >10^5^ cfu g^–1^ of FM. Compared with the control group, the content of lactic acid bacteria in the additive treatment group increased by 12.4–17.9%, and the growth of non-important bacteria was inhibited. The physiological growth of L and an adequate supply of M as a substrate could explain an increase in L count (So et al., 2020). Control silage had no satisfactory microorganisms (e.g., aerobic bacteria and yeasts), while the addition treatments dramatically reduced the counts of aerobic bacteria and molds. This was most likely due to the additive treatments’ ability to create enough lactic acid to lower pH and limit the growth of other hazardous bacteria during silage fermentation (Henderson, 1993). Ni et al. (2017) discovered that a combination of L and 2% M might prevent the growth of clostridia and enterobacter in soybean silage. Overall, these findings demonstrated that additives can lower the number of unwanted microorganisms in native grass silage, with the L + M treatment having the greatest impact.

Bacteria in fresh native grass and silage samples were sequenced using amplicon sequencing. The recovered reads retrieved from each sample ranged from 69,317 to 78,375. All of the samples had coverage values of approximately 0.99. This demonstrates that the sequencing breadth was rather broad, and the microbial high-throughput data were sufficient to define the bacterial microbial community’s features (Yang et al., 2019). It is generally recognized that these specific OTUs may have contributed to differences in silage quality (Mu et al., 2021). The OTUs, Shannon index, and Chao1 value in L + M were lower after 60 days of ensiling than in CK, L, and M. Presumably, the combined addition of L and M was more likely to the native grass fermentation, thereby inhibiting the growth of other microorganisms, because the additive treatments decreased the pH, inhibited harmful microorganisms, and promoted the growth of L species. As a consequence, the silage of the combined addition of L and M in combination had the lowest microbial diversity and richness.

The quality of silage is determined by the outcome of the competition between L and spoilage microorganisms, as well as the competition and collaboration between L (Bai et al., 2021). The PCoA plot revealed a distinct separation of bacteria in FM and additive-treated silages, indicating that ensiling rebuilds the microbial community. These results are in line with the work of Zeng et al. (2020), who also found that the bacterial communities in FM and silages were distinct. In the present study, in the early fermentation stage (7 days), the bacterial communities in the L and L + M silages were clearly separated from the other groups. This might be due to L quickly initiating lactic acid fermentation, lowering the pH, and as such impacting bacterial community succession. In addition, compared with the control treatment, the PCoA of additive treated silages was also separate, which showed that additives significantly influenced the microbial community. The variation in bacterial community might explain the difference in silage quality (Ni et al., 2017; Dong et al., 2019; Li et al., 2021). Therefore, based on the results of α diversity and beta diversity, L and M treatments could affect the microbial diversity and community structure of native grass silage.

This study found that *Proteobacteria* was the most abundant phylum in FM, accounting for more than 85%, which is consistent with previous studies that *Proteobacteria* was the dominant phylum in fresh native grass (You et al., 2021). In the present study, *Firmicutes* and *Proteobacteria* were prevalent in all treatments in our research, and the community composition altered with ensiling time. Compared with the CK group, *Firmicutes* increased, while *Proteobacteria* declined in the L and L + M groups. For maize stover and red clover silage, Xu et al. (2017) and Dong et al. (2019) found comparable findings, which might be explained by the higher microbial populations of L (You et al., 2021). *Proteobacteria* have a major role in polysaccharide utilization, organic matter degradation, and carbon cycling (Ma et al., 2018). The higher relative abundance of *Proteobacteria* in M compared with other treatments (Figure 2A) may have been due to increased fermentation substrates, which can be rapidly hydrolyzed and utilized of M by *Proteobacteria*, as was also observed by Mu et al. (2021).

In the present study, *Pantoea* was found to be the most common facultative aerobic genus in FM. *Pantoea* has been observed in fresh native grass (You et al., 2021), alfalfa (Sa et al., 2021), and soybean (Ni et al., 2017). *Pantoea* abundance decreased after 30 days of fermentation, which might be attributable to their great sensitivity to pH decline (Ogunade et al., 2018). *Lactobacillus* is a well-known microbe that decreases pH during the ensilaging process by producing lactic acid, which impacts the quality of the fermentation (Li et al., 2021). *Lactobacillus*, *Weissella*, and *Leuconostoc* are the genera with the most bacteria involved in lactic acid fermentation in silage (Yang et al., 2019), Similarly, *Lactobacillus* was the predominant genus in native grass silage treated with L + M. Ni et al. (2017) found a similar outcome when they ensiled soybean infected with L + M, and *Lactobacillus* became the dominating genus after silage fermentation was completed. *Enterobacteria* are facultative anaerobes (i.e., can live in anaerobic and acidic environments) and can metabolize WSCs and LA to produce AA, PA, and other fermentation products (Li et al., 2019; Wang Y. et al., 2019). Combined addition of M and L also contributed to the growth of *Lactobacillus*, and *Lactobacillus* abundance reached nearly 70% of the total population. It is known that lactic acid-producing cocci (*Weissella*, *Leuconostocs*, and *Lactococcus*) initiate lactic fermentation in the early ensiling process, whereas lactic acid rod (*Lactobacillus*) play a key role in pH reduction at the later stage (Cai et al., 1998). The high abundance of *Lactobacillus* in the L + M combined addition treatment could explain their relatively good fermentation quality compared with the other treatments (Ni et al., 2017; Li et al., 2021). Interestingly, in this study, the abundance of *Enterobacter* and *Escherichia* increased in M silage compared with other silages. This may be attributed to the separate addition of molasses providing a rich availability of WSC, which directly improves the competitiveness of *Enterobacter* and *Escherichia* in the silage, and thus, L could not quickly become a dominant flora. A similar trend was also observed by Mu et al. (2021). Combined addition of M and L treatments could increase the abundance of *Lactobacillus*, decrease the abundance of the *Pseudomonas*, and improve fermentation quality of native grass silage. Furthermore, their combination had a positive synergistic effect on silage fermentation and the microbial community.

Silage fermentation is a very complex biological process involving a variety of microorganisms that produces a variety of metabolites during ensiling by degrading substrates or transforming metabolites via sophisticated metabolic pathways (Guan et al., 2018). Carbohydrate, amino acid, energy and cofactor metabolism, and vitamin metabolism were found to be metabolic pathways linked to silage fermentation in previous investigations. As a result, we chose these metabolic pathways for statistical analysis, including nucleotide metabolism (Bai et al., 2021; Xu et al., 2021). In L and L + M-treated silage, carbohydrate metabolism was higher during the whole ensiling process. This might be because the addition of L accelerated the decomposition of WSC in the silage. Similarly, Bai et al. (2021) also discovered that the expression of the carbohydrate metabolism pathway was connected to the relative abundance of total L in the bacterial community. The abundance of amino acid metabolism, nucleotide metabolism, and cofactor and vitamin metabolism gradually increased with fermentation, which contradicted the findings of Sa et al. (2021) who observed that amino acid metabolism, nucleotide metabolism, and metabolism of cofactors and vitamins were decreased in the inoculated silages during the fermentation stage. This might be because the raw materials attached to L and WSC content were low, while the exogenous additives contributed to the increase in this metabolism.

## Conclusion

The present study illustrated that additives could improve the silage quality of native grass to different degrees, and that native grass silage treated with combined addition of L and M had better fermentation quality than other treatments. The use of additives prior to ensiling could reduce undesirable microorganisms and improve the nutritional value of forage native grass silage, with L + M having the best effects. In summary, the results confirmed that lactic acid bacteria and molasses exerted a beneficial synergistic effect on silage fermentation, which effectively improved silage quality, enhanced the relative abundance of *Lactobacillus*, and inhibited the growth of undesirable microorganisms on native grass.

## Data Availability Statement

The datasets presented in this study can be found in online repositories. The names of the repository/repositories and accession number(s) can be found below: https://www.ncbi.nlm.nih.gov/, PRJNA783886.

## Author Contributions

YL methodology, visualization, validation, data curation, and wrote the original draft. SD and LS interpreted the data and edited the language. QC wrote, reviewed, and edited the manuscript. GG and ZW conceptualization, acquisition of funding, and writing—reviewing and editing of the manuscript. QL and JH software. YJ conceptualization and funding acquisition. All authors have read and agreed to the published version of the manuscript.

## Conflict of Interest

The authors declare that the research was conducted in the absence of any commercial or financial relationships that could be construed as a potential conflict of interest.

## Publisher’s Note

All claims expressed in this article are solely those of the authors and do not necessarily represent those of their affiliated organizations, or those of the publisher, the editors and the reviewers. Any product that may be evaluated in this article, or claim that may be made by its manufacturer, is not guaranteed or endorsed by the publisher.
